# Inferring the basis of binaural detection with a modified autoencoder

**DOI:** 10.3389/fnins.2023.1000079

**Published:** 2023-01-26

**Authors:** Samuel S. Smith, Joseph Sollini, Michael A. Akeroyd

**Affiliations:** Hearing Sciences, Mental Health and Clinical Neurosciences, School of Medicine, University of Nottingham, Nottingham, United Kingdom

**Keywords:** binaural (two-ear) hearing effect, hearing, cross-correlation (CC), signal detection algorithm, representational learning

## Abstract

The binaural system utilizes interaural timing cues to improve the detection of auditory signals presented in noise. In humans, the binaural mechanisms underlying this phenomenon cannot be directly measured and hence remain contentious. As an alternative, we trained modified autoencoder networks to mimic human-like behavior in a binaural detection task. The autoencoder architecture emphasizes interpretability and, hence, we “opened it up” to see if it could infer latent mechanisms underlying binaural detection. We found that the optimal networks automatically developed artificial neurons with sensitivity to timing cues and with dynamics consistent with a cross-correlation mechanism. These computations were similar to neural dynamics reported in animal models. That these computations emerged to account for human hearing attests to their generality as a solution for binaural signal detection. This study examines the utility of explanatory-driven neural network models and how they may be used to infer mechanisms of audition.

## 1. Introduction

**FIGURE 1:**
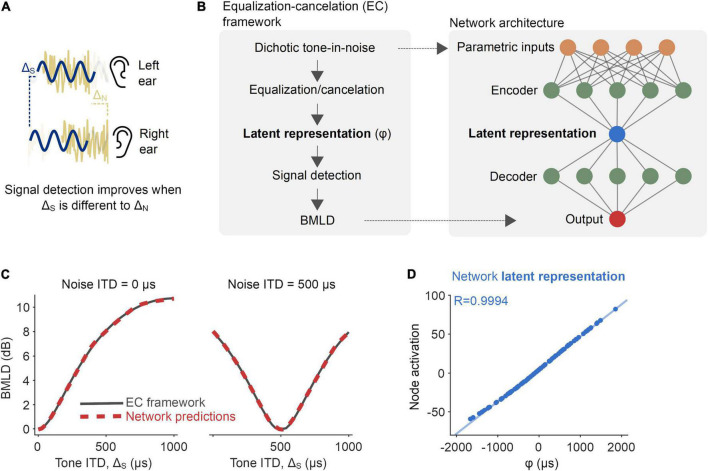
Proof-of-principle: Inferring a latent binaural variable. **(A)** The detection of a signal (sine wave denoted in navy blue) is improved if its interaural disparity is different from that of the noise (noise waveform denoted in yellow). **(B)** A neural network was trained to predict binaural masking level differences (BMLDs), as described by the equalization-cancelation (EC) framework (left). The network had a modified autoencoder architecture, in which the central layer acted as an information bottleneck. **(C)** BMLDs were numerically calculated by the EC framework (black) and estimated by the trained neural network (red-dashed), for a 500 Hz pure tone signal and noise at varying interaural time differences (ITDs). **(D)** A node central within the network had activation values entirely consistent with the latent variable as formally defined by the EC framework (φ, the signal’s post-EC ITD).

In everyday listening, it is commonplace for a sound of interest to be masked by simultaneous background sounds such as noises. If a target sound is in a different direction to a noise then they will arrive at different times to each of the ears. The auditory system takes advantage of this difference to improve the target’s detectability. In the laboratory, the prototypical method to quantify this improvement is to compare detection thresholds when (1) the signal has a different interaural time difference (ITD) to the noise, versus when (2) the signal and noise have the same ITD (Figure 1). The amount by which the former threshold is reduced in comparison to the latter is called the “binaural masking level difference” (BMLD). The value of the BMLD depends systematically on how the ITDs differ (Durlach, 1972; Durlach and Colburn, 1978) and can be as large as 15 dB at low frequencies (Hirsh, 1948; Hirsh and Burgeat, 1958). Yet, it is an open question as to what the neural mechanisms underlying human binaural detection are.

For example, midbrain and cortical recordings in non-human species lend support to a cross-correlation mechanism comparing auditory signals across the ears (Palmer and Shackleton, 2002; Lane and Delgutte, 2005; Gilbert et al., 2015). In contrast, human behavior appears to be equally well, if not better, described by a noise-cancelation scheme (Durlach, 1963; Breebaart et al., 2001a; Culling, 2007). Computational models have been built demonstrating that the cross-correlation framework and the noise-cancelation framework are both empirically feasible (Durlach, 1972; Colburn, 1977). Discrepancies between frameworks have not been resolved with human imaging data (Sasaki et al., 2005; Wack et al., 2012, 2014; Fowler, 2017), for which resolution and response variability are key limitations. As the neural activity in brain regions underlying binaural detection cannot be directly recorded in humans, we considered alternative methods of scrutiny from the field of machine learning.

The human-like “behavior” achievable with deep neural networks, combined with their unpremeditated network of computations, have seen them advocated as a new generation of model organisms (Scholte, 2018). These models can effectively approximate any mathematical function (Hornik et al., 1989), are resource efficient, relatively easy to record from and perturb activity in, and are not limited by species-specific ecology. In principle, if a network can be built that corresponds with human behavior, then knowing how that network works might give insight into the underlying human mechanisms. Yet, to date, the inner workings of neural networks configured to handle binaural audition have received limited consideration (Adavanne et al., 2018; Vecchiotti et al., 2019; Francl and McDermott, 2022), and almost exclusively in the context of binaural localization rather than detection. One potential stumbling block when interrogating the inner workings of neural network analogs is their black-box nature. However, network architectures that put mechanistic interpretability at the forefront (such as modified autoencoders that have shown promise in the field of physics; Higgins et al., 2017; Iten et al., 2018) could help overcome this.

Here, we trained neural network models to imitate the phenomena of binaural signal detection under human-like behavioral constraints, then interrogated their inner workings to discover *how* they operated. In three stages of work, we first sought validation of our methodology. Second, we developed networks that operated on waveforms to predict binaural detection performance. Third, we explored how the waveform-based networks operated, examining how they internally represented information. We discovered that not only did networks learn to make predictions similar to human behavior, but representations were found to have striking similarities with a cross-correlation mechanism similar to animal models (McAlpine et al., 1996; Lane and Delgutte, 2005; Asadollahi et al., 2010; Gilbert et al., 2015). Our key insight–that these computations emerged to account for human hearing–attests to their generality as a solution for binaural signal detection and illustrates the benefits of machine learning methods.

## 2. Results

### 2.1. Proof-of-principle: Inferring a latent binaural variable

Our goal was to use neural network models as a tool to infer computations underlying binaural detection in humans. Such an approach has proven successful in the field of physics (Iten et al., 2018). For example, in the case of predicting the movement of a pendulum, networks have correctly inferred an influential role of variables such as spring constant and damping factor. First, to demonstrate the feasibility of this methodology in the context of binaural hearing, we trained a network on a reduced example. We wanted to verify that, in the process of predicting the dynamics of a fully defined system, the network would infer the same latent variable as within said system. Accordingly, we trained networks to mimic a system of equations derived under the “equalization-cancelation” (EC) framework (Durlach, 1972, part IV.B; see Eq. 1 in Section “Materials and methods”), which is effective at reproducing the key phenomena of the detection of a pure tone signal masked by a broadband noise (Durlach, 1963; Klein and Hartmann, 1981; Breebaart et al., 2001a; Hartmann and McMillon, 2001; Culling, 2007; Wan et al., 2010). The framework proposes that the interaural configuration of the masking noise is “equalized” (=applying an internal time delay to the waveform from one ear to compensate for, or equalize for, the external temporal disparity compared to the waveform from the other ear) and “canceled” (=subtracting the equalized waveforms from one another), resulting in a more detectable signal. These EC operations give rise to a latent representation that can be captured by the variable φ (Figure 1B, left, see Eq. 1 in Section “Materials and methods” for details). In the EC framework, this variable is used to predict the consequent improvement in signal detection from binaural processing over monaural processing, i.e., BMLDs. In particular, we were interested as to whether a neural network would automatically infer the latent variable φ in the process of predicting BMLDs as described under the EC system of equations.

We trained a neural network, with a modified autoencoder architecture, to predict the *binaural* improvement in signal detection (i.e., BMLDs) based on four parameters describing the *monaural* arrival times of a 500 Hz signal and broadband noise at each ear. The input/output training data were drawn from EC equations fit to human psychophysics (Figure 1B). Following training, we tested the network on parametric combinations of BMLDs for which it had not been trained and discovered that its root-mean-square (RMS) error was just 0.075 dB. We took this as evidence that the network was able to successfully generalize its performance. The network correctly predicted larger BMLDs when the signal had a non-zero ITD and the masking noise did not, and vice versa (Figure 1C and Supplementary Figure 1A). Interrogating the computations latent within the network provided insight into how it operated. Because the network utilized a modified autoencoder architecture, its inputs were “encoded” into a simpler representation, the latent representation, by passing information through a bottleneck at the center of the network (Figure 1B, right). When we looked at the bottleneck node’s activation values (its numerical readout), we saw that its activation almost exactly matched the latent variable in the EC framework, φ (Figure 1D; Pearson’s *R* = 0.9994, *p* < 0.001), even though the model was never directly informed of that variable.

In summary, within this fully defined system, the network was able to infer the appropriate latent variable in accounting for BMLD dynamics and therefore reinforced our premise.

### 2.2. Modified autoencoder accounted for binaural detection psychophysics

In our first stage, we provided the network with four parameters quantifying a signal in a noise, whereas in reality the human auditory system would be presented with *waveforms* of a signal combined with masking noise. How these waveforms are processed as to confer a binaural advantage is an open question, nor does the EC framework make any explicit proposal about how equalization parameters would be derived from said waveforms (Durlach, 1972; Wan et al., 2010). Additionally, humans display a graded psychometric performance as signal level is varied, from an inability to full detection, for which detection thresholds only offer a single-value snapshot at one chosen performance level.

**FIGURE 2:**
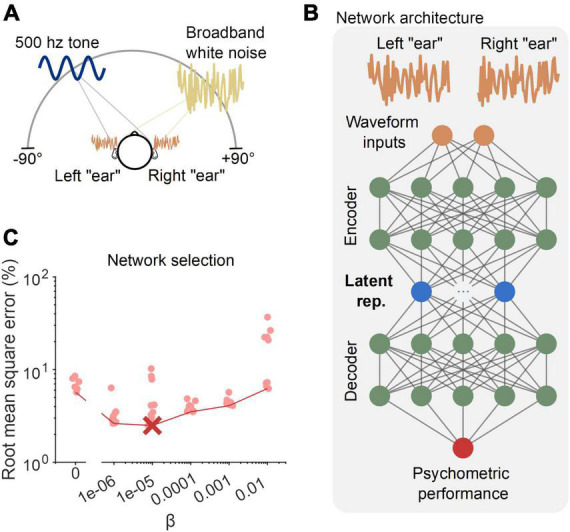
Network training and configuration. **(A)** Data from a simulated frontal field binaural detection task were used to train neural networks to detect a 500 Hz pure tone (sine wave denoted in navy blue) in broadband noise (yellow noise waveform). Locations of the tone and noise were chosen at random on each trial and were equally likely to come from each azimuthal location. **(B)** The modified autoencoder network received left/right “ear” waveforms as inputs, and had five hidden layers, with the central layer containing 10 nodes–constrained by the parameter β in their information transmission. **(C)** Error for 60 networks (10 for each value of β, see Section “Materials and methods”) tested on a held-out validation dataset. The red circles indicate the errors for the 60 networks. The red cross marks the optimally performing network, and the red line bounds the networks with minimum error for each value of β.

**FIGURE 3:**
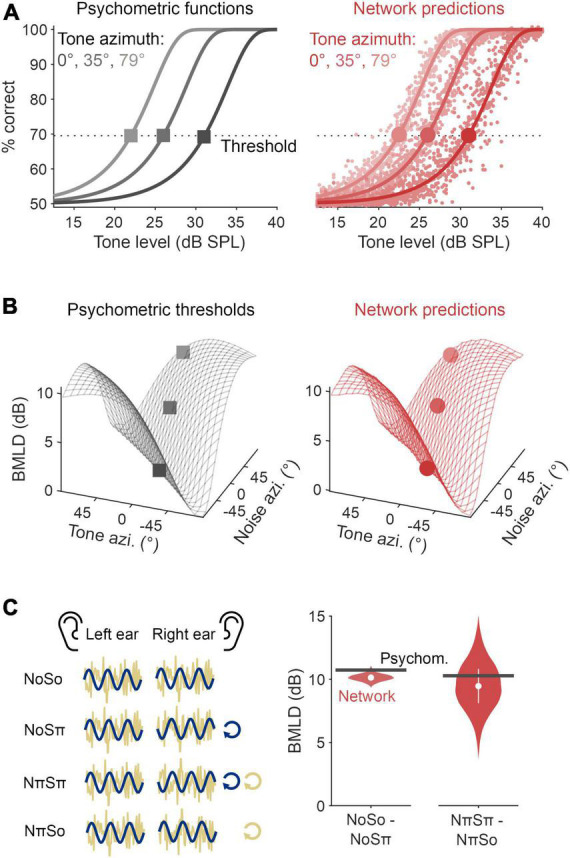
Modified autoencoder accounted for binaural detection psychophysics. **(A)** Psychometric functions quantifying tone detection as a function of tone level masked by a 60 dB SPL Gaussian noise (left, black). These functions are drawn for tones presented from three azimuths, relative to a noise presented directly in front. The optimal neural network model was able to approximate these psychometric functions (red, right), from which detection thresholds (corresponding to a d-prime of 1) and binaural masking level differences (BMLDs) could be calculated. **(B)** Psychophysical estimates (left, black) of human BMLDs for a 500 Hz tone presented in noise, each with interaural time differences (ITDs) mapped from differing azimuths. Alongside are the optimal network’s predictions (right, red). Markers representing thresholds as defined in panel **(A)** are overlaid. **(C)** (Left panel) A schematic of the laboratory stimuli configurations denoted as NoSo, NoSπ, NπSπ, and NπSo. (Right panel) BMLDs were derived for experimental stimulus configurations: NoSo/NoSπ, NπSπ/NπSo (π, for a 500 Hz signal, is beyond the range of ITDs used during training).

Accordingly, in the second part of our work, we advanced our network/training paradigm to incorporate these aspects of binaural detection. Namely, input into the networks were vectors describing waveforms simulated as arriving at the left and right “ears” (see the top of the schematic in Figure 2B). Further, networks were constrained to predict detection rates to which a graded psychometric function could be fit (see Figure 3A). We also generalized the training data to represent signals coming from random azimuthal locations in the frontal horizontal plane, restricting the range of incorporated ITDs to within an approximate human physiological range (±655 μs; Figure 2A). To generate BMLD estimates, we retained the set of equations used in Section “2.1. Proof-of-principle: Inferring a latent binaural variable” (which were fed parameters from which waveforms were constructed), as they represent good fits to human binaural psychophysics (Durlach, 1972) and augment the availability of training data. To account for the increased complexity, the autoencoder was modified to have two layers of nodes at the “encoder” and “decoder” stages and allowed for multiple (10) nodes in the central layer of the network (Figure 2B). We ran 60 separate networks, each trained on the same data, but with varying constraints as to how independently each central node represented information. This was determined by a parameter β that specified whether the emphasis was given to the predictive accuracy of the network or the interpretation and simplicity of its latent representations. This was specified within the network’s cost function, a function that specifies to what end a network should be optimized during training (see Eq. 4 in Section “5.2. Modified autoencoder network”). Based on the form of the cost function, we see that a higher value of β prioritizes the interpretation and simplicity of latent representations over predictive accuracy. Interestingly, we found that networks with a non-zero, but intermediate, value of β best accounted for a held-out set of data (Figure 2C), showing that some constraints on information encoding were better than none.

The optimal network had a root mean square error of 2.5% for the validation dataset (these networks predict detection rate, hence why the unit is % and not dB). We found this network was able to closely replicate the psychometric functions for the improvement in signal detection as the presented tone increased in level amongst a 60 dB SPL broadband noise (Figure 3A). From these data, we were able to regress functions from which to derive detection thresholds (defined as a performance level *d’* of 1) and, in turn, calculate BMLDs. We found that the network’s BMLDs increased as the difference between tone ITD and noise ITD increased (Figure 3B and Supplementary Figure 1B). For example, in diotic noise (noise ITD = 0) with a tone placed at the far left, detection thresholds were significantly enhanced by 9 dB (two-sided unpaired *t*-test, *p* < 0.001), matching human BMLD behavior (Durlach and Colburn, 1978).

To allow a comparative assessment of the neural network models and previously published work, we also presented networks with stimulus configurations typically employed in the laboratory to study binaural detection. These include tones and noise in popular laboratory configurations, either in-phase or completely out-of-phase across the ears. In the literature, these stimuli are denoted as NoSo, NoSπ, NπSπ, and NπSo, where N refers to the noise, S the pure tone signal, with the subscripts denoting interaural phase difference (IPD) in radians (see Figure 3C, left panel). Importantly, none of these stimuli were used in training, nor can most occur in everyday listening. These stimuli have ITDs that are frequency dependent and can be greater than the range permitted by head width. For example, a 500 Hz pure tone with an IPD of π corresponds to an ITD of 1,000 μs, whereas the typical value for the largest ITD due to a head is 655 μs (Woodworth et al., 1954). As our networks were trained on ITDs within the head’s range, this meant networks had no prior exposure to this magnitude of ITD and so it was unclear how they would function over this range. We found that when the noise signal had zero IPD, the BMLD for the corresponding homophasic (NoSo) and antiphasic (NoSπ) tone conditions was 10.1 dB, an effect that was statistically significant (two-sided unpaired *t*-test, *p* < 0.001). Comparatively, when instead the noise signal was interaurally out-of-phase, the predicted BMLD for the corresponding homophasic (NπSπ) and antiphasic (NπSo) stimuli was 9.5 dB, and again significant (two-sided unpaired *t*-test, *p* < 0.001). These BMLDs are similar to those typically measured in laboratory research (Durlach and Colburn, 1978) and with estimates from the psychophysical equations (10.7 and 10.3 dB, respectively; Figure 3C).

### 2.3. Latent representations imitate neural signature of population-level cortical activity

**FIGURE 4:**
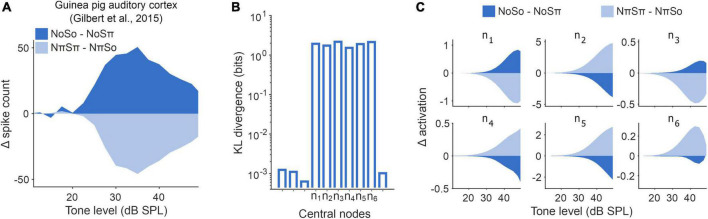
Latent representations imitated signature of population-level cortical activity. **(A)** Change in population masked rate-level functions recorded from guinea pig auditory cortex (Gilbert et al., 2015) in response to changes in experimental binaural stimuli NoSo/NoSπ (dark blue) and NπSπ/NπSo (light blue). **(B)** Kullback–Leibler (KL) divergence (Kullback and Leibler, 1951) between each individual node and a unit Gaussian. Unlabeled nodes along the x-axis were deemed to be suppressed during training. **(C)** Rate-level functions for the operational central nodes in the optimal network, comparable to panel **(A)**.

In the third stage, we investigated *how* the model achieved this behavior. To do this we, first, looked at the network’s latent representations and considered them relative to known binaural phenomena. Prior animal neural data have shown that the stimulus conditions depicted in Figure 3C (NoSo/NoSπ and NπSπ/NπSo) hint at a unique signature of binaural detection processing (Gilbert et al., 2015). In guinea pig cortical recordings, population spike counts dropped amongst a No signal as a 500 Hz tone went from So to Sπ (Figure 4A). Conversely, amongst an Nπ signal, as a pure tone transitioned from Sπ to So, population spike counts *increased*. The neural dynamics contrast, yet in both conditions binaural detection thresholds improved. We would not expect such opposing dynamics under an EC framework–a signal and a noise that are interaurally out-of-phase with one another should consistently give rise to a less “canceled” signal representation than if they were in phase with one another. Instead, this neural signature is more in line with the dynamics expected under a cross-correlation framework (demonstrated in Gilbert et al., 2015; see Section “5.9. Binaural cross-correlation algorithm” for more details on binaural cross-correlation).

We, therefore, examined the latent representation of the NoSo/NoSπ and NπSπ/NπSo stimulus conditions within the central layer of our network. To do this we needed to determine which nodes in this layer were operational, in the sense that they had non-trivial output values. We found that this was true of six nodes, whereas the remaining four had adapted to produce negligible outputs to comply with constraints on information transmission (Figure 4B). We found that the operational nodes exhibited opposing dynamics in response to the two pairs of homophasic/antiphasic stimuli (Figure 4C), although the directionality of these opposing dynamics varied across the six nodes (we believe that this is a consequence of the nodes being able to take any real number, and hence this directionality can be ignored). On average, the change in activation for NoSo/NoSπ was opposite to NπSπ/NπSo for all six operational nodes (a 2^–6^ = 0.016 chance). Although mean differences were significant (two-sided unpaired *t*-tests for tone-level of 35 dB SPL, *p* < 0.001 for all), trial-to-trial values were noisy and overlapping [two-sample K-S test between NoSo/NoSπ and NπSπ/NπSo conditions, for a tone level of 35 dB SPL, D ranged from 0.076 (n_4_) to 0.43 (n_2_), *p* < 0.001 for all (see Section “5.10. Statistical analysis”)], to be expected given the input waveforms were dominated by Gaussian noise. Some of this variance was due to the partial representation of non-binaural stimulus properties (e.g., monaural tone phase) that had not been adequately disregarded early in the network. Some of this variance could be accounted for based on the activity of other central nodes (Supplementary Figure 2A). With such co-variation accounted for, we saw a further enhanced contrast for the NoSo/NoSπ and NπSπ/NπSo stimulus conditions, markedly at threshold levels (Supplementary Figures 2B, C).

In summary, given that the network predicted similar magnitudes of BMLDs for NoSo/NoSπ and NπSπ/NπSo, *and* broadly captured opposing dynamics for these stimulus conditions, we conclude that the network imitated this key signature of binaural detection.

### 2.4. Encoder network dynamics matched those of a cross-correlator

**FIGURE 5:**
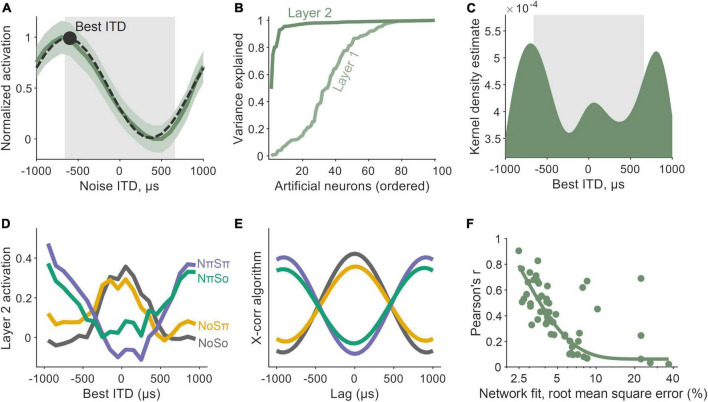
Encoder network dynamics matched those of a cross-correlator. **(A)** Interaural time difference (ITD) tuning emerged as a property of nodes within the early encoder layer of the network. The activation values of an example node are shown to vary as a function of noise ITD (dark green). Tuning was characterized by Gabor functions (black, dashed) with peaks defined as a node’s best ITD (black circle). The gray box underlays represent the ITD-limit for our training simulation. **(B)** The proportion of variance explained (R^2^) by Gabor fits, although high in Layer 1 (light green) of the encoder, was widespread by Layer 2 (darker green). **(C)** Best ITD distribution for nodes in Layer 2, characterized by a kernel density estimate (bandwidth of 200 μs). Again, the gray box underlay represents the ITD-limit for our training simulation. **(D)** Activation values of Layer 2 nodes for binaural detection stimuli: NoSo, NoSπ, NπSπ, NπSo (color-coded). Smoothed with a 600 μs moving average window. **(E)** The profiles in 5D were similar to a simple cross-correlation (X-corr) algorithm. **(F)** The better a network predicted psychophysical data (x-axis), the more similar its encoder network to a cross-correlator (y-axis).

Finally, in order to further understand the encoder network that lies between the waveform inputs and the latent representations described in the network’s central layer, we examined ITD tuning. To determine this, we computed noise delay functions in nodes within the encoding network (Figure 5A), i.e., their activation values in response to noises presented with varying ITDs. Tuning was quantified by regressing a Gabor function onto the noise-delay function (Lane and Delgutte, 2005), i.e., the combination of a cosine windowed by a Gaussian (overlaid in Figure 5A). For nodes in the encoder’s first layer, we observed significant ITD tuning in 63 out of 100 nodes (Figure 5B). By the encoder’s second layer, significant ITD tuning had emerged in all 100 nodes. Estimates of each node’s best ITD (i.e., the ITD that gives the maximum activation) were derived from Gabor fits (to account for nodes that were cyclical in their noise delay responses, the best ITD was attributed to the most central tuning peak). In both the first and second layers of the encoder network, we observed a wide distribution of best ITDs, both within the simulated head range, and beyond it (Figure 5C).

Importantly, one framework that is both commensurate with the earlier results (Section “2.3. Latent representations imitate neural signature of population-level cortical activity”) and found in animal models is that of a binaural cross-correlator mechanism (McAlpine et al., 1996; Lane and Delgutte, 2005; Asadollahi et al., 2010; Gilbert et al., 2015). The concept is predicated on the existence of coincidence detectors that encode temporally offset signals. To deduce whether our network had automatically learned to operate like a cross-correlator, we measured nodal activations in responses to the laboratory tone-in-noise conditions: NoSo, NoSπ, NπSπ, and NπSo (Figure 5D). When a signal was presented amongst an in-phase noise (No), responses were largest for nodes with best ITDs near 0 μs and decreased as best ITDs were increasingly non-zero. Conversely, amongst an out-of-phase noise (Nπ), responses were lowest for nodes with best ITDs near 0 μs and increased as best ITDs deviated away from this. The effects of the tone phase on node dynamics were more subtle, although these dynamics were also in accordance with a node’s tuning properties. Nodes tuned to smaller ITDs responded most to in-phase tones (So) and least to out-of-phase tones (Sπ), and vice-versa for nodes tuned to larger ITDs. These dynamics are consistent with a cross-correlation model.

Computationally, a binaural cross-product can be calculated by summing the point-by-point product of two temporally offset signals. Comparative outputs from a simple binaural cross-correlation algorithm (namely for signals in noise passed through narrow-band filters centered at 500 Hz) are shown in Figure 5E. We saw a significant correlation between the network and the cross-correlation calculation (with local averaging: Pearson’s *r* = 0.91, *p* ≪ 0.001; without: Pearson’s *r* = 0.36, *p* ≪ 0.001). When looking across all 60 of the networks that we trained, we found that the more a network made predictions that matched the psychophysical data, the more similar its encoder network was to a cross-correlator (Figure 5F).

## 3. Discussion

Binaural detection of a signal masked by noise is a well-standardized laboratory measurement that underpins important theories of auditory processing. However, the underlying mechanisms involved remain uncertain. Here, we used machine learning methods to infer potential mechanisms underlying human-like binaural detection. We found that our neural networks were able to successfully utilize interaural discrepancies across dichotic signal-in-noise waveforms to predict human-like binaural detection behavior. Notably, similarities with animal neural dynamics and a binaural cross-correlator were emergent within the network. We emphasize that these dynamics were not hard-coded into the network, they were learned, and highlight their importance in the context of signal detection, not just the more commonly referenced function of sound localization (Joris and Yin, 2007). These findings promote the understanding of how neural network models operate as an effective tool for investigating the basis of binaural processing.

### 3.1. The basis of binaural detection

In our study, we utilized a set of equations originally derived under the assumptions of the EC framework (Durlach, 1972), treating them as accurate numerical fits to human binaural psychophysical data (see Section “5.1. Binaural detection rates and thresholds”). This is the case, and was in part the motivation, for the experimental parameters investigated in this study [i.e., the EC framework fits well to human psychophysics for a 500 Hz tone and ITDs, but not for ILDs (Wan et al., 2010)]. Yet, our findings overall support a different process for interaural detection, namely, cross-correlation. The distinction is important because Domnitz and Colburn (1976) provided statistical evidence that, under certain assumptions, models based on temporal or phase differences (as the EC framework is) provide similar predictions of tone-in-noise detection to interaural correlation-based models. They concluded that comparing binaural detection predictions made by both classes of models is insufficient to disentangle underlying mechanisms. To circumvent this, we inverted the conventional forward-approach to modeling, and instead reverse engineered our models. We discovered that our models developed a cross-correlation mechanism to reproduce psychophysical data. We also observed that central nodes broadly reproduced opposing dynamics to NoSo/NoSπ and NπSπ/NπSo, consistent with population neural activity in animal models. In contrast, one would expect that an EC-like noise cancelation scheme would operate similarly for both NoSo/NoSπ and NπSπ/NπSo stimulus conditions, and hence would not exhibit these opposing dynamics. Further, we found that additional mechanisms that utilize *a priori* knowledge of the masker, as have been proposed for some EC models (Hawley et al., 2004), are not required in order to account for binaural detection behavior. Taken together, one interpretation of our findings is that, in its analytical form, the EC framework captures the “computational goal” of the system (Marr and Poggio, 1976), enacted *via* means of a binaural cross-correlator. An alternative interpretation is that, although binaural cross-correlation produced a sufficient decision variable for the detection of simple stimuli, for more complex tasks and stimuli (e.g., speech recognition), binaural cross-correlation could instead be used to derive optimal delay parameters within a hybrid EC framework (Culling, 2020).

Despite the occurrence of the earlier mentioned network dynamics, the model exhibited flaws including dynamics that were less tangible. For example, we observed instances in which central nodes partially represented seemingly irrelevant stimulus properties, e.g., monaural phase. As opposed to the encoder network filtering out these stimulus properties, the network appeared to separately represent this co-variation and account for it at a later stage. This is possibly a consequence of the modified autoencoder architecture’s preference for capturing separate latent variables in separate nodes (Higgins et al., 2017; Iten et al., 2018), potentially augmented by an over-resourced “decoder” network. In addition to these divergent dynamics, for some extreme stimulus configurations, we observed some slight discrepancies in predicted and ground truth detection thresholds, although we stress that relative differences (i.e., BMLDs) were accurately predicted. We trained our models on stimuli with ITDs limited by a typical head size (i.e., ±655 μs). However, there is evidence that natural sound statistics can incorporate ITDs beyond this limit (Młynarski and Jost, 2014). Training networks on such distributions may improve the predictive performance for extreme stimulus configurations.

We note that we have modeled only a fraction of the BMLD conditions that have been experimentally tested (see Breebaart et al., 2001a,b,c; Bernstein and Trahiotis, 2017). It will be of interest to learn how far a model like ours can further generalize to other parametric laboratory stimuli. Potential tests range from confirming more standard results such as the effect of the interaural correlation of the noise (Robinson and Jeffress, 1963; van der Heijden and Trahiotis, 1997; Bernstein and Trahiotis, 2020) to exploring results that apparently require extensions such as longer delay lines (van der Heijden and Trahiotis, 1999, but see Encke and Dietz, 2022 and Eurich et al., 2022, for an opposing interpretation). Given that our model is essentially a “stationary signal” model, at minimum an extended set of training stimuli would likely be necessary for detecting dynamically changing signals, such as those demonstrating “binaural sluggishness” (Kollmeier and Gilkey, 1990).

### 3.2. Neural network analogs of auditory processing

Understanding of binaural detection in humans has been mired due to ambiguity regarding whether animal neurophysiology data satisfactorily accounts for human psychophysics. Whilst not a substitution for “ground-truth” neurophysiology, treating deep neural networks as a model organism (Scholte, 2018) appears to be a promising approach to bridging together neural and behavioral data. Recent neural network studies have described correlates with broad organizational principles in the auditory system (Kell et al., 2018; Koumura et al., 2019; Khatami and Escabí, 2020) and asked questions of “why” a neural system operates in a particular way. Here, we focused on the question of “how” a system operates, for the well characterized binaural phenomena of improved detection of a 500-Hz tone in noise. Despite the notable computational similarities between our trained networks and neural observations, comparisons between neural network models and neural biology come accompanied by an asterisk. The network was not constructed with the goal of accurately mimicking neuronal biophysics or hierarchical complexity, but instead a trade-off was made in which a modified autoencoder architecture (Iten et al., 2018) was applied to facilitate interpretation and optimization. In future work, the limits of this network architecture could be further examined and improved by considering how predicted BMLDs are influenced by spectral and temporal qualities of the masker and target signals (Breebaart et al., 2001b,c). Further scaling of this modeling approach, for example, to examine interaural level differences or across-frequency integration, would also likely be insightful. However, any impact on interpretability should be weighed (even in this, arguably simplified, context the network dynamics were non-trivial), and such models are first contingent on the generation of suitably large psychophysical datasets.

## 4. Conclusion

In conclusion, our results newly demonstrate that neural network models, utilizing a modified autoencoder architecture, can discover key computations underlying binaural hearing. Latent activity within the model corroborates observations made in animal physiology and speaks to their generality as a solution to binaural detection. The work demonstrates the potential for machine learning methods to help bridge the gap between neurophysiology and psychophysics.

## 5. Materials and methods

### 5.1. Binaural detection rates and thresholds

The framework of equalization and cancelation (Durlach, 1972) has human psychophysical support, accurate in predicting binaural masking level difference (BMLD) data (Durlach, 1972), binaural pitch phenomena (Durlach, 1972; Klein and Hartmann, 1981; Hartmann and McMillon, 2001), and underpinning other models of binaural hearing (Breebaart et al., 2001a). Although psychophysical predictions made under this framework do not extend to individual differences, they are sufficient to consider presumed commonalities across individuals. Numerical predictions of BMLDs in decibels were calculated from phenomenological equations derived from this framework (Durlach, 1972; Wan et al., 2010):


(1) $$\text{BMLD}\,\left(\tau_{S},\tau_{N}\right)=10\,\log_{10}\max\left\{\frac{k-\cos\left(\omega_{0}\,\varphi \right)}{k-\gamma \,\left(\tau_{N}-\tau_{0}\right)},1\right\}$$

where τ_*S*_ and τ_*N*_ are the interaural time lags of the signal and noise, ω_0_ is the angular frequency of the pure tone signal, $k=\left(1+\sigma_{\varepsilon }^{2}\right)\,e^{\omega_{0}^{2}\,\sigma_{\delta }^{2}}$ where $\sigma_{\varepsilon }^{2}$ and $\sigma_{\delta }^{2}$ are jitter (internal noise) parameters with the values proposed by Durlach ($\sigma_{\varepsilon }^{2}=0.25$ and $\sigma_{\delta }^{2}$ = 105 μs), γ is the normalized envelope of the autocorrelation of the narrow-band noise output of a triangular-gain filter centered at the target tone frequency, and τ_0_ is an optimal time equalization parameter. The parameter φ = τ_*S*_−τ_*N*_ represents the difference in interaural time of the tone and noise signals (in Section “2.1. Proof-of-principle: Inferring a latent binaural variable” we examined whether a neural network could discover this parameter). The values of the other parameters were chosen according to Durlach’s (1972) original formulation in which the model was fit to human data.

Psychometric functions were derived from BMLDs calculated in Eq. 1 (Egan et al., 1969), with detection thresholds defined as equivalent to a *d’* of 1 in a yes-no experiment (Green and Swets, 1966):


(2) $$\mathrm{detection}\,\mathrm{rate}=100\,\Phi \,\left(\frac{m_{0}\,10^{0.1\,\left(\text{BMLD}+a-23\right)}}{2}\right)$$

where BMLD is from Eq. 1, *a* is pure tone pressure amplitude in decibels, and Φ is the cumulative normal distribution. Assuming a nominal diotic detection threshold of 31 dB SPL, we can solve for *m_0_*:


(3) $$m_{0}=\frac{2\,\Phi^{-1}\,\left(0.69\right)}{10^{0.8}}\approx 0.16$$

where *a* is 31 dB SPL, BMLD is 0 dB, and detection rate is 69 %.

### 5.2. Modified autoencoder network

We ran autoencoder-based, three-part neural network models (Higgins et al., 2017; Iten et al., 2018). The three parts are the encoder, central, and decoder layers. Networks took input values that were passed through exponential linear unit (ELU) layer(s), referred to as the “encoder” portion of the network. This was followed by a single central layer with Gaussian node(s) (≥the number of parameters varied in the generation of training stimuli) with minimal uncorrelated representations, constrained by a parameter β which balances network regularization versus network interpretation. This was followed by further ELU layer(s), referred to as the “decoder” portion of the network. All layers were fully connected and feed forward. The Adam optimization algorithm (Kingma and Ba, 2014) was used to minimize the cost function:


(4) $$C_{\beta }\,\left(\hat{x},x,\sigma ,\mu \right)={||\hat{x}-x||}_{2}^{2}-\frac{\beta }{2}\,\sum_{i}\log\left(\sigma_{i}^{2}\right)-\mu_{i}^{2}-\sigma_{i}^{2}$$

where $\hat{x}$ and *x* are predicted and ground truth outputs, respectively (subscript 2 is the L2 norm, superscript 2 is squaring), σ and μ are the standard deviation and mean of Gaussian nodes, respectively, and the *i* subscripts reference separate central nodes. Architecture meta-parameters were influenced by those described in Iten et al. (2018). Network weights and biases were randomly initialized. The number of training instances employed in each iterative update of network parameters (i.e., the batch size) was set to 256. The learning rate (training hyperparameter) was set to 5 × 10^–4^ for 1,000 epochs (i.e., total passes of the entire training dataset).

### 5.3. Parameter-based network

The first “proof-of-principle” network (see Section “2.1. Proof-of-principle: Inferring a latent binaural variable”) took four parametric inputs representing the arrival times of each of a 500 Hz pure tone and broadband noise at each ear. The network was trained to predict BMLDs as specified in Eq. 1. The network was trained and validated (95%/5% split, respectively) on 100 000 instances of monaural tone and noise arrival times, each randomly drawn from between 0 and 2,000 μs. The encoder and decoder portions each had one layer with 100 ELU nodes. The central layer had two nodes (one was suppressed during training) with β set to 10^–5^.

### 5.4. Waveform-based networks

In our second model (Sections “2.2. Modified autoencoder accounted for binaural detection psychophysics” to “2.4. Encoder network dynamics matched those of a cross-correlator”) we trained networks using waveforms of a signal combined with masking noise. In this way, and in contrast to the “proof-of-principle” network, individual stimulus characteristics were not initially known by the system. These networks took 800 input values, representative of simulated left ear and right ear waveforms, each of 400 samples as simulated from a pure tone and noise mapped to different angles in the azimuth. Networks were trained to predict the corresponding detection rates, as specified in Eq. 2. Training/validation (95%/5% split, respectively) was performed with 1,000,000 instances of a random phase tone in randomly generated white noise. Pure tones had 10 periods, completing one period per 40 samples. Pure tones were treated as 500 Hz for generating estimates in Eq. 1. Pure tones were set to levels between 0 and 50 dB SPL. Pure tones were masked by randomly distributed broadband noise (50–5,000 Hz, limited by 6th order Butterworth bandpass filter) with an overall level of 60 dB SPL. The tone and noise were gated simultaneously. Tones and noises were simulated with ITDs mapped from two independent angles in the azimuth between −90° (far left) and 90° (far right). ITDs were derived from Woodworth’s equation (Woodworth et al., 1954), assuming a head radius of 0.0875 m. Based upon this formula and waveform sampling, the azimuth had an effective resolution between 5.6° and 10.3°, depending upon the region within it.

The encoder and decoder portions of the network each had two 100-neuron ELU layers. The central layer of networks had 10 Gaussian nodes. As the optimization of artificial neural networks was non-deterministic, and we wished to derive a network representative of a global minimum, ten networks were trained for each value of β, namely, 0,10^–6^, 10^–5^, 10^–4^, 10^–3^, and 10^–2^ giving 60 in total. The network with the least root mean square error between predicted detection rates and ground truth for the validation dataset was selected for further analysis. Central nodes were considered operational if the Kullback–Leibler divergence (Kullback and Leibler, 1951) between their individual responses and a unit Gaussian was larger than 0.1 bits.

### 5.5. An example network calculation

We illustrate the computations from input-to-output in the waveform-based networks described in Section “5.4. Waveform-based networks” and schematized in Figure 2B. First, a weighted sum is performed on the input vector (representative of the left/right ear waveforms) and this is passed through a non-linear function. Formally, *f*(*x*) = a(W^*T*^*x*), where *x* is the input vector, W^*T*^ is a vector of trainable weights (incorporating a bias term), and a() is the non-linear “activation” function defined as:


(5) $$\text{a}\,\left(z\right)=\left\{ \begin{array}{cc} z, & z\geq 0\\ e^{z}-1, & z<0 \end{array}\right..$$

This computation gives us the “activation value” for one artificial neuron (also referred to as a node). This process is repeated 100 times, once for each of the 100 neurons in the layer–where each neuron has its own unique set of weights. We effectively have a multivariate function between the network inputs and the first layer of neurons. This transform is then repeated where the outputs of the first layer of neurons become the inputs to the next. Ultimately, we end up with 100 activation values corresponding to the number of neurons in the final layer of the “encoder.” Separate weighted sums of these 100 values are computed to represent mean and standard deviation parameters describing ten latent Gaussian distributions. These parameters form the basis of the information bottleneck of the autoencoder. These parameters are used to generate 10 randomly sampled values, μ_*i*_ + σ_*i*_ϵ, where μ_*i*_ and σ_*i*_ are the mean and standard deviation parameters defining the *i*-th latent Gaussian distribution, and ϵ∼N(0,1) a random normally distributed number. These randomly drawn values are then used as inputs to the “decoder” network. The computations of the “decoder” mimic the “encoder,” but with separately defined weights, and with one final weighted sum output–the predicted binaural detection performance. For more thorough details on the modified autoencoder architecture, please see Iten et al. (2018).

### 5.6. Network predictions

Binaural masking level difference (BMLD) predictions were generated by averaging outputs for 10 repeats of a given stimulus configuration (i.e., stimulus ITDs would be fixed whilst other parameters were randomized 10 times). For the waveform-based networks, BMLDs had to be derived based on detection rates. To determine detection thresholds, the mean of 10 detection rates for tone levels, set between 0 and 50 dB SPL in 2.5 dB SPL steps, were regressed with a psychometric curve (Eq. 2; Figure 3A). BMLDs were predicted for (i) random phase tones amongst randomly generated broadband noise with ITDs each mapped from fixed azimuthal locations spaced between ±90° (corresponding to the effective resolution, namely, 0°, ±5.61°, ±11.27°, ±16.97°, ±22.76°, ±28.67°, ±34.73°, ±41.01°, ±47.56°, ±54.45°, ±61.80°, ±69.77°, ±78.60°, and ±88.71°), and (ii) random phase tones amongst randomly generated broadband noise each either in- or out-of-phase (i.e., NoSo, NoSπ, NπSπ, and NπSo).

### 5.7. Artificial neural representations

Artificial neuron activation values (=a node’s numerical expression) were measured in response to the stimuli configurations in Section “5.6. Network predictions.” Activation values were also measured as a function of ITD for broadband noise only (50–5,000 Hz, 60 dB SPL). ITDs ranged from −2,000 to 2,000 μs in steps of 100 μs. For the parametric-based network, central layer activation values were measured in response to 100 random stimulus generations. For the waveform-based networks, activations were measured in response to 5,000 random stimulus generations.

### 5.8. ITD tuning

Interaural time difference (ITD) tuning was quantified by fitting a Gabor function (Lane and Delgutte, 2005) to noise delay responses. The parametric expression for a Gabor function is:


(6) $$G=A\,e^{-{\left(I\,T\,D-b\,I\,T\,D\right)}^{2}/2\,s^{2}}\,\cos\left(2\,\pi \,F\,\left(I\,T\,D-b\,I\,T\,D\right)\right)+C$$

in which we characterized a node’s best ITD as the parameter *bITD*, *F* is the tuning curve frequency, *A* is a scaling factor (constrained to be positive), *C* is a constant offset, and *s* is a decay constant. These parameters were fit with the non-linear least squares algorithm curve_fit in SciPy (Virtanen et al., 2020). An *F*-test was used to assess whether a Gabor function was a significantly better fit to noise delay responses than a linear function of ITD.

### 5.9. Binaural cross-correlation algorithm

For comparative purposes, we ran a standard psychophysical model of binaural cross-correlation (Akeroyd, 2017). It produced an output approximating an ensemble of neurons rather than individual spike trains. The stimuli NoSo, NoSπ, NπSπ, and NπSo were generated for a 35 dB SPL tone and a 60 dB SPL randomly distributed broadband noise (the algorithm utilized computer representations of dB SPL). Stimuli were sampled at 20 kHz and were 1 s in duration. Signals were passed through gammatone filters centered at 500 Hz and passed through a non-linear model of neural transduction (Meddis et al., 1990). The outputs were then delayed relative to one another, and the cross-products were calculated and summated.

### 5.10. Statistical analysis

We performed Student’s two-tailed *t*-tests (assuming unequal variance) to assess differences between BMLDs. Pearson product-moment correlation was calculated between the average responses of nodes to NoSo, NoSπ, NπSπ, and NπSo, and the delay matched outputs of a binaural cross-correlation algorithm (see Section “5.9. Binaural cross-correlation algorithm”). Correlations were calculated with, and without, local averaging (within 600 μs). Student’s two-tailed *t*-tests (assuming unequal variance) and two-sample Kolmogorov–Smirnov tests were performed to compare changes in central node activation values for homophasic/antiphasic stimuli pairs. The D statistic of the Kolmogorov–Smirnov test is the absolute maximum distance between the cumulative distribution functions of the two samples. The *p*-value returned by the Kolmogorov–Smirnov test is the probability that the null hypothesis, that two samples were drawn from the same distribution, is rejected. For the outlined statistical analyses, the criterion for significance was set to *p* = 0.05. Violin plots were used to capture data probability density in Figure 3C and Supplementary Figure 2. The lightly shaded underlay in Figure 5A shows standard errors. In Figure 5F an exponential curve was robustly fit with the least absolute residual method.

## Data availability statement

The main materials presented in this study can be found in online repositories. The names of the repository/repositories and accession number(s) can be found below: https://github.com/Hearing-Sciences/BinauralDetection_DNN.

## Author contributions

SS and MA: conceptualization. SS: methodology, investigation, and writing – original draft. SS, JS, and MA: interpretation and writing – review and editing. MA: funding acquisition, resources, and supervision. All authors contributed to the article and approved the submitted version.

## Supplementary material

Supplementary Figure 1 **(A)** The prediction error between the equalization-cancelation (EC) framework and network predictions in Figure 1C. **(B)** The prediction error between the EC framework and network predictions in Figure 3B.

Supplementary Figure 2 **(A)** Some central nodes orthogonally represented stimulus-properties. For example, n_3_ sinusoidally varied in activation value as a function of monaural tone phase. Shown for NoSo with tone level at 20 dB SPL. **(B)** Near threshold (tone level of 20 dB SPL), the distribution of values when comparing the change in n_1_ activation between NoSo/NoSπ (dark blue, left) and NπSπ/NπSo (light blue, right) are considerably overlapping. Two-sample KS test statistic, D, is 0.11, *p* < 0.001. **(C)** When the co-variate captured by n_3_ is controlled for (e.g., looking at when n_3_ < 0, i.e., monaural tone phase between 0 and π) the distinction between the conditions is clearer. Two-sample KS test statistic, D, is 0.8, *p* < 0.001.
