# Supplementary figures and images for: Inferring the basis of binaural detection with a modified autoencoder
Source: Front Neurosci. 2023 Jan 26;17:1000079. doi: 10.3389/fnins.2023.1000079 (PMC9909603; doi:10.3389/fnins.2023.1000079)

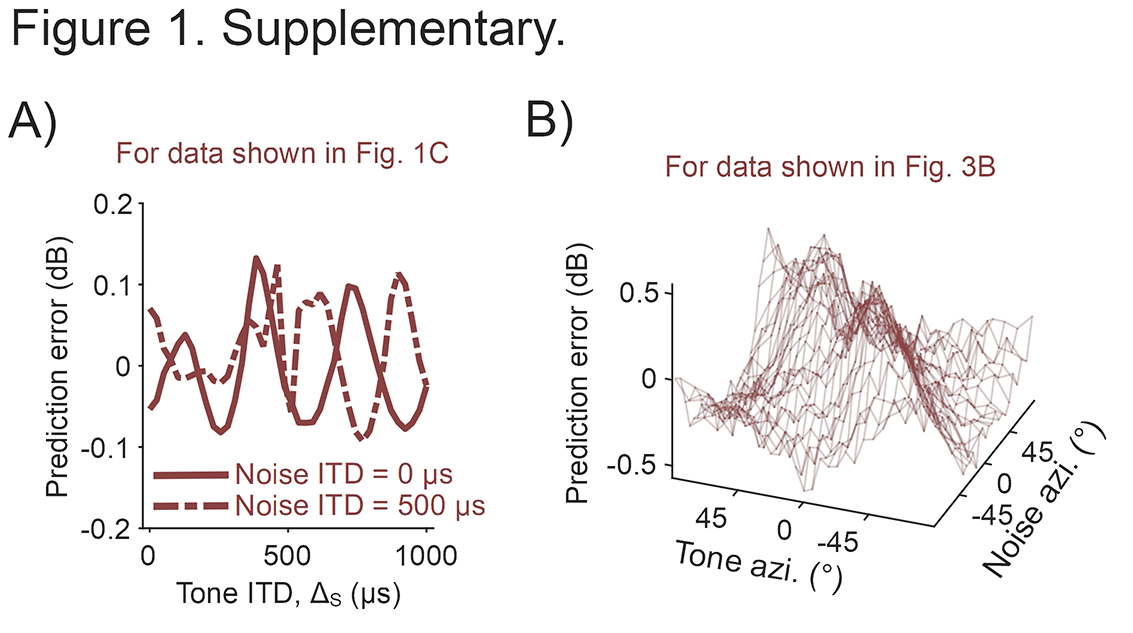

Supplement: Supplementary Figure 1 — (A) The prediction error between the equalization-cancelation (EC) framework and network predictions in Figure 1C. (B) The prediction error between the EC framework and network predictions in Figure 3B. [file Image_1.TIFF]

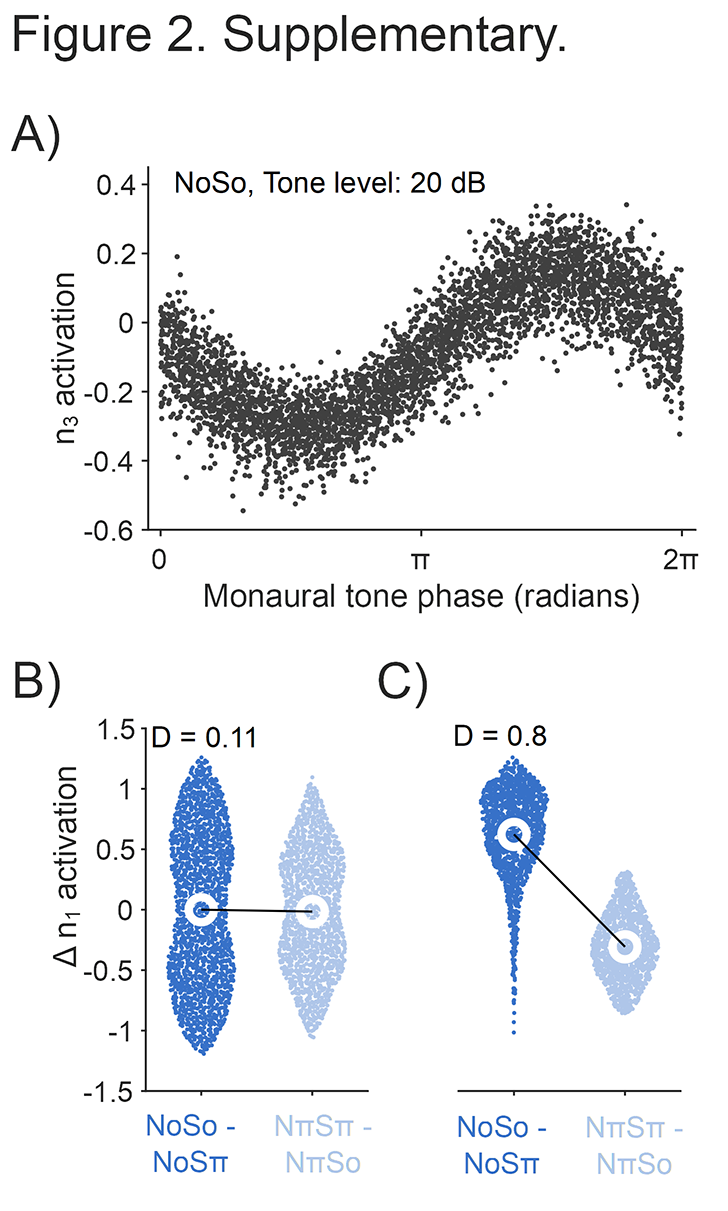

Supplement: Supplementary Figure 2 — (A) Some central nodes orthogonally represented stimulus-properties. For example, n3 sinusoidally varied in activation value as a function of monaural tone phase. Shown for NoSo with tone level at 20 dB SPL. (B) Near threshold (tone level of 20 dB SPL), the distribution of values when comparing the change in n1 activation between NoSo/NoSπ (dark blue, left) and NπSπ/NπSo (light blue, right) are considerably overlapping. Two-sample KS test statistic, D, is 0.11, p < 0.001. (C) When the co-variate captured by n3 is controlled for (e.g., looking at when n3 < 0, i.e., monaural tone phase between 0 and π) the distinction between the conditions is clearer. Two-sample KS test statistic, D, is 0.8, p < 0.001. [file Image_2.TIFF]
